# The relationship between cognitive function and cortical thickness in first-episode drug-naive schizophrenia patients with agitation

**DOI:** 10.3389/fpsyt.2025.1576215

**Published:** 2025-04-24

**Authors:** Qian Liang, Yan Li, Chao Zhou, Rongrong Zhang, Shuiping Lu, Xuran Shen, Fuli Jiang, Shiping Xie

**Affiliations:** Department of Psychiatry, The Affiliated Brain Hospital of Nanjing Medical University, Nanjing, China

**Keywords:** agitation, cognitive function, cortical thickness, schizophrenia, MRI

## Abstract

**Objective:**

This study aims to explore the relationships between the agitation behavior, cognitive function and cortical thickness in first-episode drug-naïve schizophrenia (FESN).

**Methods:**

A total of 55 male healthy controls (HC) and 79 male inpatients with FESN were enrolled in the present study. Whole brain cortical thickness was extracted from T1-weighted MRI using Freesurfer Version 7.4.1 software package. Cognitive function was evaluated using the MATRICS Consensus Cognitive Battery (MCCB). The Positive and Negative Syndrome Scale-Excited Component (PANSS-EC) is used to divide these inpatients into agitation group (FESN+A) and non-agitation group (FESN+NA). Correlation analysis was employed to investigate the potential associations between cortical thickness and cognitive function.

**Results:**

The FESN+A group had higher Positive and Negative Syndrome Scale (PANSS) total score, positive symptom score, and general psychopathology score than the FESN+NA group. Both the FESN+A/NA groups showed significantly worse performance than the HC in symbol coding, working memory, attention/vigilance, reasoning and problem solving, and social cognition. The FESN+A group performed worse on working memory when comparing to FESN+NA group. Furthermore, the cortical thickness of the left paracalcarine gyrus was increased in the FESN+NA group, compared to HC. FESN+A group had thicker cortical thickness in the right posterior cingulate cortex (rPCC) compared with the FESN+NA group. The cortical thickness of rPCC was negatively correlated with score of working memory in the FESN+A group.

**Conclusion:**

The present study demonstrated that the abnormal cortical thickness of rPCC may be related to the agitation behavior and cognitive function in patients with FESN+A, suggesting a potential treatment target for agitation behavior and cognitive impairment in schizophrenia.

## 1 Introduction

Schizophrenia (SZ) is a complex and chronic mental disorder with a global prevalence of approximately 1% (1). Its core clinical features encompass positive symptoms, negative symptoms and cognitive impairments (2). SZ frequently results in severe disability, impaired social functioning, and a poor long-term prognosis (3). Additionally, patients may exhibit behavioral abnormalities, such as agitation. Agitation, also referred to as excitement symptoms, manifests as excessive emotional, behavioral, and cognitive hyperactivity in patients with SZ. In more severe cases, these symptoms can escalate to extreme behaviors including hostility, verbal threats, impulsive actions, aggressive violence, and even self-harm. The risk of violent behavior in patients with SZ is 1 to 7 times higher than in healthy controls (HC) (4). Such agitation behaviors may have a significant impact on the cognitive function and brain structure of patients, though their relationship remains not fully elucidated.

SZ patients with cognitive impairment frequently exhibit significant deficits in working memory, verbal memory, visual memory, processing speed, and executive function, which can severely impact daily life and social adaptation, potentially leading to poor prognosis and long-term disability (5, 6). Research has demonstrated a significant correlation between neurocognitive decline and an increased risk of violent behavior in SZ patients (7). For example, Elena et al. found that SZ patients who engaged in violent behavior performed significantly worse on symbol encoding tasks compared to nonviolent SZ patients (8). Ahmed et al. identified working memory, reasoning/problem solving, language learning, and executive dysfunction as significant predictors of aggressive behavior in SZ patients (9, 10). Social cognitive impairment also plays a crucial role in the violent behavior of SZ patients. Previous studies have shown that empathy deficits modulate aggressive behavior in chronic SZ patients (11). Moreover, SZ patients with a history of violent behavior exhibited significant impairments in facial emotion recognition compared to those without such a history (12). In SZ patients, the attribution affected by severe delusions is a critical factor in predicting paranoid thoughts and is significantly associated with aggressive behavior (13). These studies suggest that agitation may be linked to cognitive dysfunction in SZ patients. However, most of these studies focused on chronic SZ patients and those with long-term medication, leaving the specific effects of medication and disease duration unverified. First-episode drug-naïve schizophrenia (FESN) patients, free from medication interference and early in their disease course, provide an ideal population for investigating the relationship between agitation symptoms and cognitive function.

Numerous studies have demonstrated abnormal changes in brain structure among patients with SZ, which may be closely associated with an increased risk of impulsive aggressive behavior. These alterations broadly involve multiple key brain regions, including the frontal and temporal lobes, the limbic system, and the cerebellum (14–16). Recent research has identified a correlation between violent behavior and microstructural abnormalities in several brain areas in SZ patients, specifically within the frontal lobe, uncinate fasciculus, and superior longitudinal fasciculus (17, 18). Patients with SZ who exhibit violent behavior show significantly reduced gray matter volume (GMV) in the left frontal pole, bilateral hippocampal, inferior temporal gyrus, middle temporal gyrus, temporal pole, fusiform gyrus, and insula, compared to those nonviolent behavior (19–21). Furthermore, GMV in the hypothalamic region was found to be diminished when compared to HC and exhibited a negative correlation with scores on both the Positive and Negative Syndrome Scale (PANSS) and Modified Overt Aggression Scale among violent SZ patients (22). In addition to GMV findings, studies focusing on cortical thickness have revealed that SZ patients with a history of violence demonstrate significantly reduced cortical thickness in the right inferior parietal gyrus, sensorimotor areas, and ventrolateral prefrontal cortex when contrasted with nonviolent patients (19, 23, 24). Moreover, Baumann et al. reported a positive correlation between the cortical thickness of the frontal cortex and impulsive behaviors observed in patients diagnosed with SZ (25). These structural brain abnormalities related to cognitive function and emotional regulation underscore complex interconnections between brain changes, cognitive dysfunction, and agitation within patients with SZ. However, research on the relationship between agitation behavior and cortical thickness specifically within FESN remains relatively limited.

To conduct a precise analysis of the relationship between agitation symptoms, cognitive function, and cortical thickness, this study scanned the structural MRI and assessed the Positive and Negative Syndrome Scale-Excited Component (PANSS-EC) and the MATRICS consensus cognitive battery (MCCB) in patients with FESN. The hypothesis of this study is that abnormal cortical thickness in specific brain regions may be associated with agitation behaviors and cognitive function in patients diagnosed with FESN.

## 2 Materials and methods

### 2.1 Participants

Considering that male patients in this population have a higher risk of violent behavior (7), a total of 79 male patients with FESN were recruited consecutively from the inpatient department in the Affiliated Brain Hospital of Nanjing Medical University between November 2021 and June 2024. All patients were diagnosed consistently by two experienced senior psychiatrists according to the Diagnostic and Statistical Manual of Mental Disorders–V criteria (DSM-V). After at least 6 months of follow-up, all patients enrolled in the study were eventually diagnosed with SZ. Inclusion criteria for all SZ patients were as follows: (1) Han ethnicity, right-handed, age between 16 and 45; (2) education years ≥ 7 years, intelligence quotient (IQ) ≥ 70; (3) first episode illness, duration of their first experience of psychosis ≤ 24 months, no taking antipsychotic medications and no physical therapies; (4) the score of 60 or more on the PANSS. Exclusion criteria encompassed significant physical health issues, organic mental disorders, dementia or intellectual disability, along with any history of substance abuse.

55 male HC were recruited from the social channels via poster advertisements. The HC were screened using the Structured Clinical Interview for DSM-IV-TR Axis I, non-Patient Edition (SCID-I/NP), and met the following conditions: Han ethnicity, right-handed, age between 16 and 45; no personal history of psychosis, or a family history of mental disorder. The exclusion criteria were the same as the patients group.

The study was approved by the Medical Research Ethics Committee of the Affiliated Brain Hospital of Nanjing Medical University. All participants provided written informed consent.

### 2.2 Clinical assessments

The age, gender, years of education and handedness were obtained from the patients and their parent or guardian as the demographic information. The Chinese version of the Wechsler Adult Intelligence Scale-Revised (WAIS) was applied for measuring the IQ, which included four sub-tests: the common sense, similarity, picture completion tests, and block design. The PANSS was used for psychopathological assessment in SZ patients, which involved positive symptom, negative symptom and general psychiatric symptom. Specifically, we will adopt the PANSS-EC to assess agitation symptoms, which include Excitement (P4), Hostility (P7), Tension (G4), Non-cooperation (G8), and Poor Impulse Control (G14). Patients were divided into the FESN with agitation (FESN+A) group using the PANSS-EC total scores ≥ 14 and one or more items scores ≥ 4. The FESN with non-agitation (FESN+NA) group was defined as the patients without FESN+A during the first episode (26). We ultimately enrolled 48 FESN+A patients and 31 FESN+NA patients to participate in the study.

### 2.3 Measures of cognitive function

The MCCB was used to evaluate the cognitive function in all participants (27), which consisted of nine subitems: trail making test (TMT), Hopkins verbal learning test–revised (HVLT-R), brief visuospatial memory test–revised (BVMT-R), category fluency: animal naming (Fluency), Mayer-Salovey-Caruso emotion intelligence test: managing emotions (MSCEIT ME), brief assessment of cognition in schizophrenia: symbol coding (BACS SC), Wechsler memory scale-III: spatial span (WMS-III SS), neuropsychological assessment battery: mazes (NAB Mazes), and continuous performance test-identical pairs (CPT-IP). A total of nine tests scores were matched with age, gender and years of education by MCCB transfer software to obtain T scores (28).

### 2.4 Magnetic resonance imaging data acquisition

All participants underwent MRI on a 3.0 T Siemens Verio magnetic resonance imaging scanner (Erlangen, Germany). T1-weighted MPRAGE structural MRI scans took the following optimized acquisition parameters: repetition time (TR) = 2530 ms, echo time (TE) = 2.98 ms, inversion time (TI) = 1100 ms, flip angle = 7°, voxel size = 1.0mm×1.0mm×1.0 mm, matrix size = 256×224×192, slice thickness = 1.00 mm, field of view (FOV) = 256mm×256 mm. During the scan, participants should keep awake, eyes closed, head fixed, supine position quietly, and not perform specific cognitive tasks. Wear the earplugs to avoid scanner noise and reduce head motion.

### 2.5 Cortical thickness measurement

The T1-weighted images were processed using the FreeSurfer Version 7.4.1, an open neuroimaging toolkit, to automatically acquire measurement of cortical thickness in each hemisphere. Initially, T1-weighted images underwent registration that included correcting signal in homogeneity and applying affine transformation for image correction; non-brain tissues such as the scalp and skull were subsequently removed. The software’s built-in algorithm then automatically identified and segmented the subcortical structures, standardized the image signal intensity, separated the white matter from gray matter portions, and constructed the cortical surface of both types of matter. Finally, smoothing was applied at the gray-white matter interface while expanding the cortical surface for enhanced visualization and analysis purposes. The software simulated how pia mater expands outward toward cerebrospinal fluid boundaries to measure cortical thickness based on the Desikan-Killiany-Tourville (DKT) Atlas which divides the cerebral cortex into two hemispheres with 34 cortical regions (29).

### 2.6 Statistical analysis

Data analysis was carried out using SPSS 27.0 software. For demographic and clinical data evaluation, we employed the Shapiro-Wilk test to check for normality in data distribution; one-way Variance model (ANOVA) was used for normally distributed data while non-parametric tests addressed those not conforming to a normal distribution pattern. Additionally, Pearson correlation analysis explored relationships between cognitive function and cortical thickness. A p-value of less than 0.05 was considered statistically significant. The *p*-values were corrected with Benjamini-Hochberg (BH) and Bonferroni, and corrected *p*-values of less than 0.05 were considered to indicate statistical significance.

## 3 Results

### 3.1 Demographic and clinical characteristics

The demographic and PANSS scores are shown in **Table 1**. No significant age differences emerged among the three groups (H=2.742, *p*>0.05). In addition, the years of education showed statistically meaningful variation in three groups(H=20.023, *p*<0.001), and the HC group had more years of education than the FESN+A(corrected *p*=0.002) and FESN+NA groups(corrected *p*<0.001), but there was no significant difference among the FESN subgroups(corrected *p*>0.05). The FESN+A group had higher total scores (t=2.615, *p<*0.05), positive symptom scores (Z=-2.072, *p<*0.05), and general psychopathology scores (Z=-4.500, *p*<0.05) than the FESN+NA group. There was no significant difference in scores on the negative symptom score domain (Z=-0.847, *p*>0.05).

**Table 1:** Demographic data and clinical assessment of FESN+A/NA and HC.

	FESN+A (n=48)	FESN+NA (n=31)	HC (n=55)	H/t/Z	*P*
Age (years)	25.00 (19.25,30.75)	26.00 (18.00,32.00)	24.00 (22.00,33.00)	2.742	0.254 <0.001***
Years of Education	13.00 (11.00,15.75)	12.00 (11.00,15.00)	15.00 (15.00,16.00)	20.023	
Handedness (right/left)	48/0	31/0	55/0	NA	NA
PANSS Total symptom	90.63±7.272	86.65±5.401	NA	2.615	0.011*
Positive symptom	25.50 (23.00,27.00)	23.00 (22.00,26.00)	NA	-2.027	0.043*
Negative symptom	19.00 (13.25,22.00)	19.00 (16.00,22.00)	NA	-0.847	0.397
General psychopathology	47.00 (45.00,49.00)	44.00 (43.00,45.00)	NA	-4.500	<0.001***

Medians (quartiles) represent data that are not normally distributed. Mean ± standard deviation (SD) represents that the data were normally distributed. FESN+A, first-episode drug-naive schizophrenia patients with agitation. FESN+NA, first-episode drug-naive schizophrenia patients with non-agitation; HC, healthy controls; PANSS, Positive and Negative Syndrome Scale. * *p*<0.05, *** *p*<0.001.

### 3.2 Cognitive function

**Figure 1:**
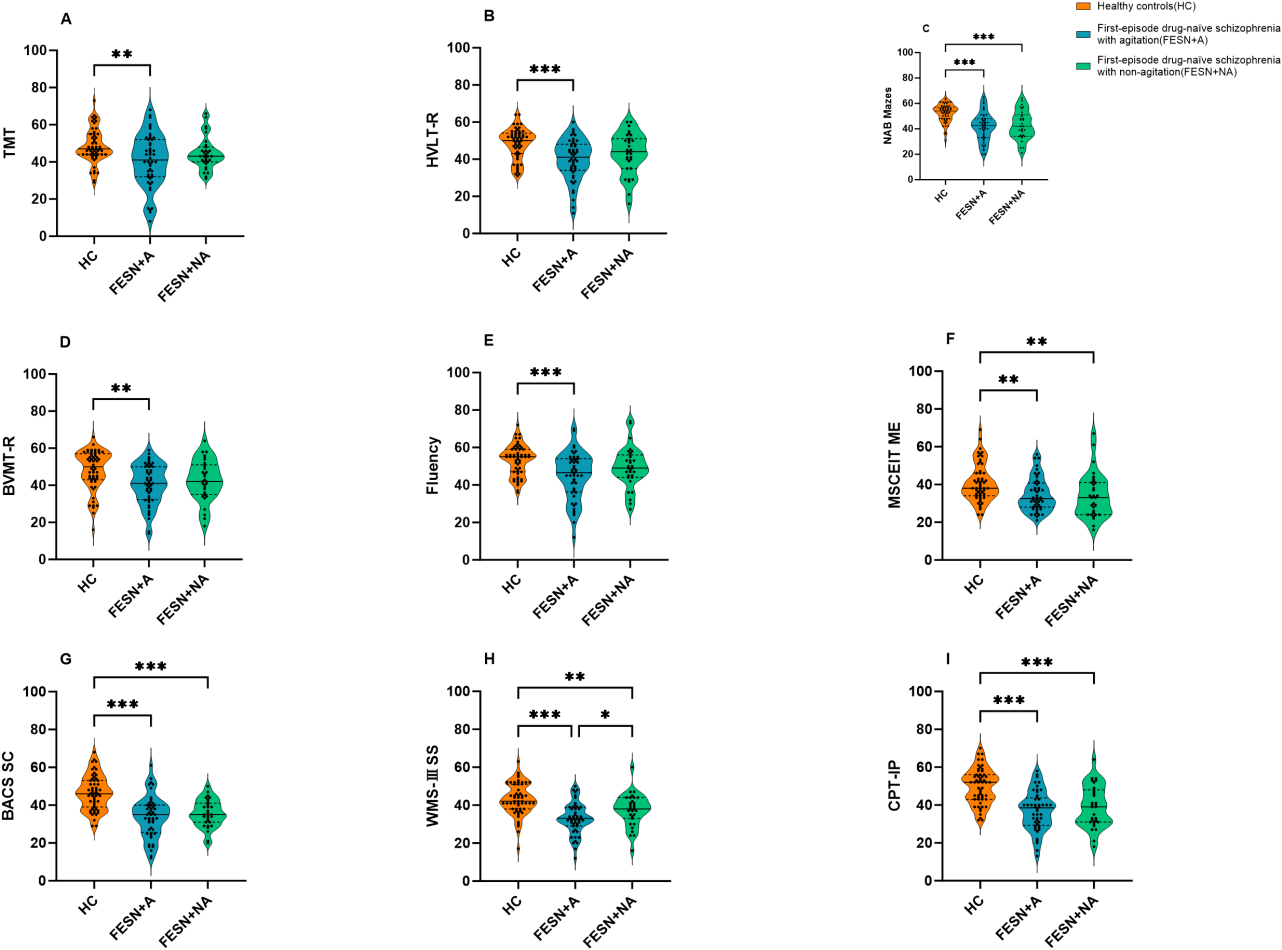
The differences in cognitive function among the HC, FESN+A group and FESN+NA group. **(A-F)** The performance of the three groups on TMT, HVLT-R, NAB Mazes, BVMT-R, Fluency and MSCEIT ME are shown. The violin plot shows the distribution of the data, including the quartiles. The *p* values were corrected by Bonferroni. **(G-I)** The performance of the three groups on BACS SC, WMS-III SS and CPT-IP are shown. The *p* values corrected by BH (Benjamini-Hochberg). The center line of each violin plot indicates the median and the width indicates the data density. Corrected *p* values of less than 0.05 were considered statistically significant, and the level of significance in the figure is indicated by asterisks: *p < 0.05, **p < 0.01, ***p < 0.001. FESN+A: agitated patients with first-episode drug-naïve schizophrenia. FESN+A, first-episode drug-naive schizophrenia patients with agitation. FESN+NA, first-episode drug-naive schizophrenia patients with non-agitation. HC, healthy controls; TMT, Trail Making Test; HVLT-R, Hopkins Verbal Learning Test - Revised; BVMT-R, Brief Visuospatial Memory Test - Revised; Fluency, Category Fluency, Animal Naming; MSCEIT ME, Mayer - Salovey - Caruso Emotion Intelligence Test, Managing Emotions; BACS SC, Brief Assessment of Cognition in Schizophrenia, Symbol Coding; WMS-III SS, Wechsler Memory Scale - III, Spatial Span; NAB Mazes, Neuropsychological Assessment Battery, Mazes; CPT-IP, Continuous Performance Test - Identical Pairs.

All the scores of cognitive tasks in FESN group were significantly lower than that of HC (all *p*<0.01), as shown in **Table 2**. In the comparisons among FESN+A/NA groups and HC, both FESN+A/NA groups showed lower scores in symbol coding, working memory, attention/vigilance, reasoning, problem-solving, and social cognition, compared to HC (all corrected *p*<0.01). In addition, compared to HC, patients with FESN+A group performed worse in trail-making test (H=10.288, corrected *p*<0.01), word learning (H=15.958, corrected *p*<0.001), visual learning (H=14.155, corrected *p*<0.01), and fluency (H=14.131, corrected *p*<0.01). Patients with FESN+A performed worse than those FESN+NA in working memory (F=18.841, corrected *p*<0.05, Cohen’s *d*=-0.531), but there were no significant differences in the other domains of cognitive function between these two groups (all corrected *p*>0.05) (**Figure 1**).

**Table 2:** Cognitive function in FESN and HC.

	FESN (n=79)	HC (n=55)	t/Z	*P*	Cohen’s *d*
TMT	43.00 (35.00,50.00)	47.00 (44.00,55.00)	-3.137	0.002**	NA
HVLT-R	43.00 (35.00,50.00)	50.00 (43.00,54.00)	-3.781	<0.001***	NA
BVMT-R	42.00 (34.00,50.00)	50.00 (43.00,57.00)	-3.681	<0.001***	NA
Fluency	48.00 (41.00,54.00)	55.00 (47.00,59.00)	-3.497	<0.001***	NA
MSCEIT ME	33.00 (27.00,41.00)	38.00 (34.00,46.00)	-3.742	<0.001***	NA
BACS SC	34.61±9.651	46.73±9.170	7.297	<0.001***	1.282
WMS-III SS	34.68±8.611	43.07±8.410	5.601	<0.001***	0.984
NAB Mazes	42.14±11.111	52.42±7.060	6.542	<0.001***	1.064
CPT-IP	37.86±10.595	49.89±9.345	6.781	<0.001***	1.191

FESN, patients with first-episode drug-naïve schizophrenia; HC, healthy controls; TMT, Trail Making Test; HVLT-R, Hopkins Verbal Learning Test – Revised; BVMT-R, Brief Visuospatial Memory Test – Revised; Fluency, Category Fluency: Animal Naming. MSCEIT ME, Mayer - Salovey - Caruso Emotion Intelligence Test: Managing Emotions. BACS SC, Brief Assessment of Cognition in Schizophrenia: Symbol Coding. WMS-III SS, Wechsler Memory Scale - III: Spatial Span. NAB Mazes, Neuropsychological Assessment Battery: Mazes. CPT-IP, Continuous Performance Test - Identical Pairs. Medians (quartiles) represent data that are not normally distributed. Mean ± standard deviation (SD) represents that the data were normally distributed. ** *p*<0.01, *** *p*<0.001.

### 3.3 Cortical thickness

**Figure 2:**
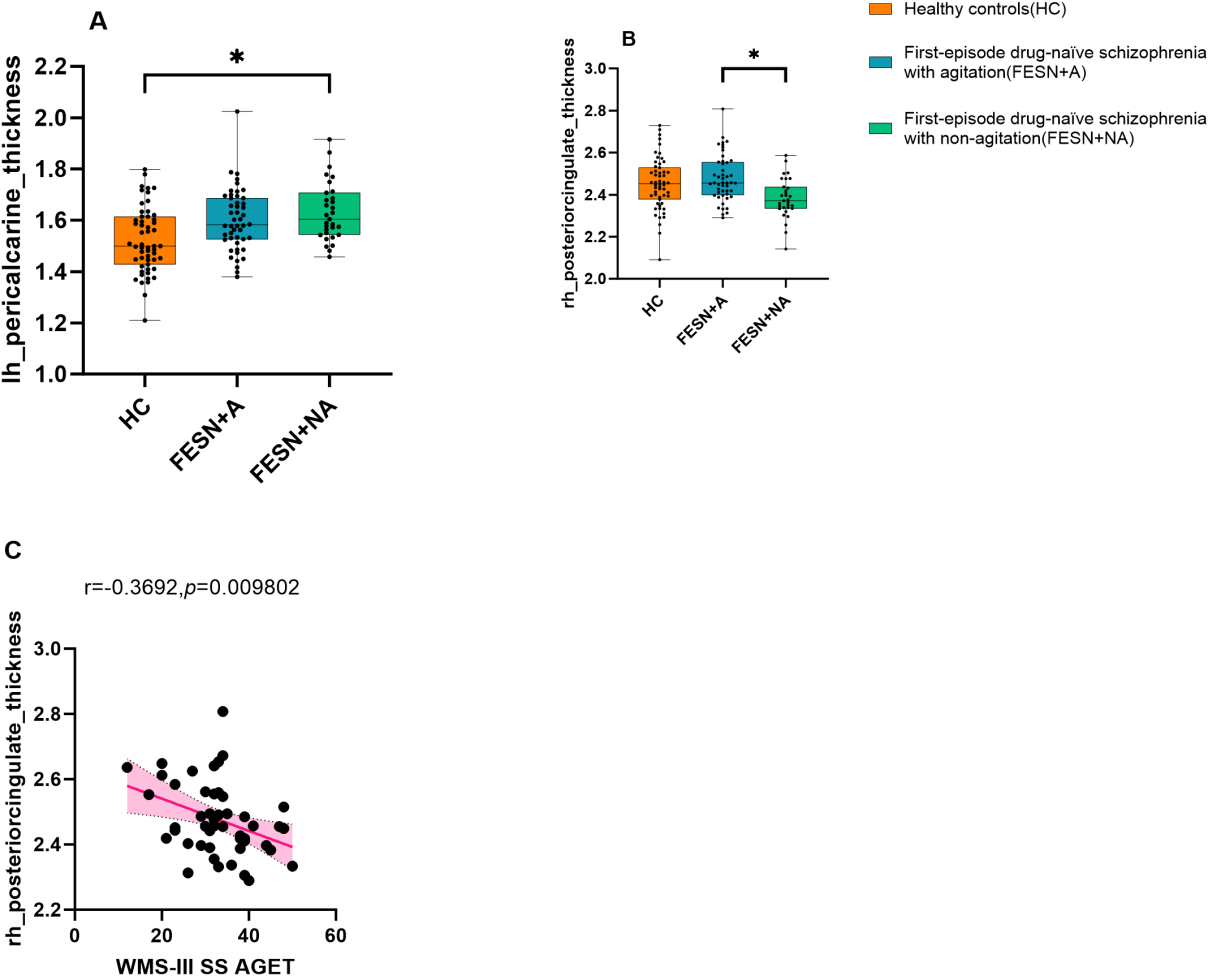
The cortical thickness variations among the three groups and the correlation between working memory and the cortical thickness of the rPCC in the FESN+A group. **(A-C)** The performance of the three groups on the cortical thickness of the left pericalcarine gyrus and right posterior cingulate cortex are shown. The *p* values corrected by BH (Benjamini-Hochberg). The box boundaries from bottom to top represent the first quartile (Q1), median (Q2), and third quartile (Q3), respectively. The “whiskers” outside the box extend to the minimum and maximum values in the data range, but do not include outliers. Corrected *p* values of less than 0.05 were considered statistically significant, and the level of significance in the figure is indicated by asterisks: *p < 0.05. FESN+A, first-episode drug-naive schizophrenia patients with agitation. FESN+NA, first-episode drug-naive schizophrenia patients with non-agitation; HC, healthy controls; rPCC, right posterior cingulate cortex.

Compared with HC, FESN group showed increased cortical thickness in bilateral paracalcarine gyrus (all corrected *p<*0.05), as shown in **Table 3**. Furthermore, FESN+NA group had increased cortical thickness in left pericalcarine gyrus compared to HC (F=9.428, corrected *p<*0.05). There was no significant difference in the cortical thickness of this region between FESN+A group and HC (F=9.428, corrected *p*>0.05). FESN+A group showed a significant thickening of cortical thickness in the right posterior cingulate cortex (rPCC) compared with FESN+NA group (F=6.717, corrected *p<*0.05, Cohen’s *d*=0.898) (**Figure 2**).

**Table 3:** The different cortical thickness of FESN and HC.

	FESN (n=79)	HC (n=55)	T	*P’*	Cohen’s *d*
left pericalcarine gyrus	1.61±0.12	1.52±0.13	-4.190	0.002**	-0.736
right pericalcarine gyrus	1.61±0.13	1.53±0.15	-3.328	0.039*	-0.584

FESN, patients with first-episode drug-naïve schizophrenia; HC, healthy controls. Mean ± standard deviation (SD) represents that the data were normally distributed. * *p’*<0.05, ** *p’*<0.01 (be corrected by B-H).

### 3.4 Correlation analysis

Pearson correlation analysis showed that the cortical thickness of rPCC was negatively correlated with score of working memory in FESN+A group (r=-0.37, *p*=0.010, **Figure 2**). No correlation was found between the cortical thickness of rPCC and the score of working memory in FESN+NA group (*p*>0.05).

## 4 Discussion

This study investigated the differences in brain structure and cognitive function between FESN+A/NA groups and HC, and further explored the relationships between cortical thickness, cognitive function, and agitation symptoms in FESN. Our main findings are as follows: Firstly, compared with HC, both FESN+A/NA groups showed cognitive dysfunction, and the FESN+NA group showed a significant increase in cortical thickness in the left paracalcarine gyrus. In addition, compared with the FESN+NA group, the FESN+A group showed more severe working memory impairment, and the cortical thickness of the rPCC was significantly increased in the FESN+A group. Finally, working memory is negatively correlated with the cortical thickness of the rPCC in FESN+A patients. To our best knowledge, this study is the first to explore the impact of agitation symptoms on cognitive dysfunction and cortical thickness abnormalities in FESN patients.

In this study, cortical thickness changes in the rPCC were found to be significantly different only within the FESN subgroup. This study reveals that increased cortical thickness in the rPCC may specifically refer to a neurobiological feature of agitation symptoms in patients with SZ rather than an intrinsic component of the schizophrenic pathological process. The PCC, located in the parico-occipital junction region of the brain, is the core of the limbic system and default mode network (DMN) and is essential for cognitive functions such as spatial perception, memory, emotion, and problem solving (30). PCC dysfunction may increase SZ risk (31). Decreased PCC GMV was observed in patients with SZ and their siblings, and non-psychotic populations with aggressive or antisocial behavior also have decreased volume in limbic regions associated with executive function, including the PCC (32, 33). In light of the inconsistency between our findings and those of prior studies, we hypothesize that this discrepancy may be associated with neuroinflammation, neurodevelopmental abnormalities, and compensatory mechanisms. Cui et al. proposed that short-term brain structural changes in first-episode SZ patients might be linked to the transcription levels of inflammatory factors. Their study demonstrated that cortical thickness changes in first-episode patients were positively correlated with monocyte gene expression (34). Meanwhile, Cheng et al. identified that a higher monocyte/high-density lipoprotein ratio could predict aggressive behavior during the acute phase of schizophrenia (35). Consequently, the increased cortical thickness of the rPCC observed in our study may reflect an early inflammatory response in SZ patients. Hoang et al.’s research corroborates our hypothesis, as they reported increased thickness in the right parahippocampal gyrus and other cortical regions among first-episode SZ patients with high inflammation levels compared to those with low inflammation levels. However, no significant differences in clinical symptoms were detected between these two groups in their findings (36). Therefore, additional investigations are warranted to validate this association. It is important to note that our study focused exclusively on FESN patients, who are less likely to be influenced by long-term neurodegenerative processes. In contrast, Zhou et al.’s findings indicated that the PCC volume progressively decreases with disease progression and worsening negative symptoms (37). Thus, the rPCC thickening observed in our study may also be attributed to the shorter disease duration of the enrolled participants. Additionally, the phenomenon of increased cortical thickness in our study may represent a critical component of the neuroadaptive mechanisms in the early stages of schizophrenia, aligning closely with the “neural compensation hypothesis.” Furthermore, previous studies revealed the abnormally enhanced activation in PCC may be associated with agitation in patients with SZ (38, 39). Although direct evidence confirming a causal link between PCC dysfunction and agitation is lacking, PCC dysfunction may indirectly contribute to impulsive aggressive behavior by affecting emotional processing, cognitive function, and neurotransmitter balance. For example, abnormal PCC activation in self-reflection and non-introspective tasks in SZ patients may distract attention, affect decision-making, and induce agitation or aggressive behavior (40, 41). Furthermore, functional dysconnectivity in PCC is associated with violent behaviors. The connectivity between the ventromedial prefrontal cortex and the PCC is impaired in patients with antisocial behavior (33). The enhanced functional connectivity between hippocampus and PCC may be related to introspective thinking in DMN (42). Based on the above findings, abnormal PCC connectivity may cause irrational responses under impulse. But hype-connectivity in the PCC was found to be associated with higher arousal and extroverted attention in SZ patients, possibly reducing attentional impulsivity (43). Neurotransmitter imbalance in the PCC is also associated with agitation behavior. Low expression of monoamine oxidase is associated with reduced cingulate cortex volume, affecting reactive aggression and psychiatric traits (44). Previous studies have also found that the level of pro-acetylcholine is increased in PCC of SZ patients, which may be associated with impulsive behavior (45). Meanwhile, the relationship between PCC and agitation is complex and needs further study.

Previous studies have found that cognitive dysfunction is particularly prominent in multiple domains in violent SZ patients, especially in working memory and verbal learning (9). These cognitive deficits are considered prime predictors of patients’ risk of future violent behavior. Our study also revealed that the FESN+A group performed worse on the working memory than the FESN+NA group. Working memory impairment is a persistent cognitive deficit in patients with SZ, which extends from the initial stage of the disease to all stages of the disease course (46). In addition, our further analysis revealed a significant negative correlation between working memory and cortical thickness of the rPCC in the FESN+A group. This correlation may point to a potential link between abnormal PCC region function and working memory impairment. The PCC generally experiences activity inhibition when performing working memory tasks (47). Existing studies have shown that reduced functional connectivity of the PCC within the DMN is positively correlated with working memory performances in patients with SZ (48). Alternatively, a lower degree of inactivation of the PCC was associated with poorer working memory performance in patients with SZ (49). These findings provide a new theoretical basis and potential intervention targets for the clinical treatment and intervention strategies of SZ.

In our study, patients in the FESN+NA group showed a significant increase in cortical thickness of the left paracalcarine gyrus compared with HC. In addition, there were no significant differences in the performance of trail making tests, word learning, visual learning and fluency between these patients and HC. However, according to the existing neurodevelopmental studies, the volume, surface area and thickness of the cerebral cortex increase with age in early childhood, while the cortical structure decreases due to the initiation of cortical apoptosis in late childhood and adolescence (50). Our findings imply that the increased cortical thickness in the left paracalcarine gyrus may indicate a neurobiological adaptive change to symptoms in patients with FESN. Based on earlier neuroimaging findings, aberrant enhanced activation of the ventrolateral prefrontal cortex has been observed in patients with early-onset SZ during a 2-back working memory task. This activation pattern may reflect a compensatory neural mechanism designed to counter the impairment of working memory to achieve cognitive performance matching that of HC (51). These results provide support for a correlation between structural changes in specific brain regions and coping strategies with cognitive difficulties in patients with SZ.

However, this study faces some inherent limitations. First, the cross-sectional design of this study limits the longitudinal tracking of patient changes over time, which limits the establishment of causal relationships between variables. In addition, this study did not adequately control for a potential confounding variable, education level, which could have had a significant impact on the findings. Future studies should adopt longitudinal study designs while strictly controlling for potential confounders to more precisely explore the dynamic interactions among agitation symptoms, cognitive function, and cortical thickness, as well as their associations with disease progression and treatment response. To provide a stronger scientific basis for tailored cognitive rehabilitation programs for patients with SZ. Additionally, the sample of this study was limited to male patients, a limitation that limits the generalizability and extrapolation of the findings. Therefore, future study designs should include female patients in order to improve the representativeness of the findings. Our study was limited by an inadequate sample size, which may have increased the risk of false negative results. Furthermore, the present study relied solely on the scores of the PANSS-EC for the assessment of agitation symptoms, which has certain limitations. Subsequent studies should incorporate multimodal assessment tools to allow detailed analysis of the associations between agitation symptoms, cognitive function, and structural changes within the cortex.

## 5 Conclusions

In conclusion, in this study, patients in the FESN+A group suffered more severe cognitive impairment in the working memory domain than those in the FESN+NA group, and the cortical thickness of the rPCC in the FESN+A group was significantly thicker than that in the FESN+NA group. Furthermore, there is a negative correlation between cortical thickness of the rPCC and working memory performance in the FESN+A group. Taken together, our preliminary findings suggest that the abnormal cortical thickness of rPCC may be related to the agitation behavior and cognitive function in patients with FESN+A. These preliminary results need to be verified in future studies with more rigorous experimental design and methodology. Meanwhile, exploring the neurobiological mechanisms underlying these associations is also a key direction for future research.

## Data Availability

The raw data supporting the conclusions of this article will be made available by the authors, without undue reservation.

## References

[1] Liu Y Fu X Tang Z Li C Xu Y Zhang F. Altered expression of the CSMD1 gene in the peripheral blood of schizophrenia patients. BMC Psychiatry. (2019) 19:113. doi: 10.1186/s12888-019-2089-4 30987620 PMC6466712

[2] Kim JY Kong CH Kim DY Min JW Park K Jeon M. Effect of D-pinitol on MK-801-induced schizophrenia-like behaviors in mice. Phytother Res. (2023) 37:5904–15. doi: 10.1002/ptr.v37.12 37654104

[3] Bipolar Disorder and Schizophrenia Working Group of the Psychiatric Genomics Consortium. Genomic dissection of bipolar disorder and schizophrenia, including 28 subphenotypes. Cell. (2018) 173:1705–1715.e16. doi: 10.1016/j.cell.2018.05.046 29906448 PMC6432650

[4] Fazel S Gulati G Linsell L Geddes JR Grann M. Schizophrenia and violence: systematic review and meta-analysis. PloS Med. (2009) 6:e1000120. doi: 10.1371/journal.pmed.1000120 19668362 PMC2718581

[5] Shao T Huang J Zhao Y Wang W Tian X Hei G. Metformin improves cognitive impairment in patients with schizophrenia: associated with enhanced functional connectivity of dorsolateral prefrontal cortex. Transl Psychiatry. (2023) 13:315. doi: 10.1038/s41398-023-02616-x 37821461 PMC10567690

[6] Fleischhacker WW Podhorna J Gröschl M Hake S Zhao Y Huang S. Efficacy and safety of the novel glycine transporter inhibitor BI 425809 once daily in patients with schizophrenia: a double-blind, randomised, placebo-controlled phase 2 study. Lancet Psychiatry. (2021) 8:191–201. doi: 10.1016/S2215-0366(20)30513-7 33610228

[7] Yi Y Huang Y Chen Q Yang H Li H Feng Y. Violence, neurocognitive function and clinical correlates in patients with schizophrenia. Front Psychiatry. (2022) 13:1087372. doi: 10.3389/fpsyt.2022.1087372 36741559 PMC9893505

[8] Gobbi E Cotelli M Manenti R Ferrari C Macis A Bianconi G. Neuropsychological features in patients with severe mental disorders and risk of violence: A prospective multicenter study in Italy. Psychiatry Res. (2020) 289:113027. doi: 10.1016/j.psychres.2020.113027 32417593

[9] Ahmed AO Richardson J Buckner A Romanoff S Feder M Oragunye N. Do cognitive deficits predict negative emotionality and aggression in schizophrenia? Psychiatry Res. (2018) 259:350–7. doi: 10.1016/j.psychres.2017.11.003 29120842

[10] Serper M Beech DR Harvey PD Dill C. Neuropsychological and symptom predictors of aggression on the psychiatric inpatient service. J Clin Exp Neuropsychol. (2008) 30:700–9. doi: 10.1080/13803390701684554 18608673

[11] Bragado-Jimenez MD Taylor PJ. Empathy, schizophrenia and violence: a systematic review. Schizophr Res. (2012) 141:83–90. doi: 10.1016/j.schres.2012.07.019 22917950

[12] Bulgari V Bava M Gamba G Bartoli F Ornaghi A Candini V. Facial emotion recognition in people with schizophrenia and a history of violence: a mediation analysis. Eur Arch Psychiatry Clin Neurosci. (2020) 270:761–9. doi: 10.1007/s00406-019-01027-8 31106387

[13] Pinkham AE Harvey PD Penn DL. PARANOID INDIVIDUALS WITH SCHIZOPHRENIA SHOW GREATER SOCIAL COGNITIVE BIAS AND WORSE SOCIAL FUNCTIONING THAN NON-PARANOID INDIVIDUALS WITH SCHIZOPHRENIA. Schizophr Res Cognit. (2016) 3:33–8. doi: 10.1016/j.scog.2015.11.002 PMC5156478 27990352

[14] Gou N Lu J Zhang S Liang X Guo H Sun Q. Structural deficits in the frontotemporal network associated with psychopathic traits in violent offenders with schizophrenia. Front Psychiatry. (2022) 13:846838. doi: 10.3389/fpsyt.2022.846838 35492688 PMC9039223

[15] Kumari V Uddin S Premkumar P Young S Gudjonsson GH Raghuvanshi S. Lower anterior cingulate volume in seriously violent men with antisocial personality disorder or schizophrenia and a history of childhood abuse. Aust N Z J Psychiatry. (2014) 48:153–61. doi: 10.1177/0004867413512690 24234836

[16] Puri BK Counsell SJ Saeed N Bustos MG Treasaden IH Bydder GM. Regional grey matter volumetric changes in forensic schizophrenia patients: an MRI study comparing the brain structure of patients who have seriously and violently offended with that of patients who have not. BMC Psychiatry. (2008) 8 Suppl 1:S6. doi: 10.1186/1471-244X-8-S1-S6 18433516 PMC2330074

[17] Hoptman MJ Volavka J Johnson G Weiss E Bilder RM Lim KO. Frontal white matter microstructure, aggression, and impulsivity in men with schizophrenia: a preliminary study. Biol Psychiatry. (2002) 52:9–14. doi: 10.1016/S0006-3223(02)01311-2 12079725

[18] Waller R Dotterer HL Murray L Maxwell AM Hyde LW. White-matter tract abnormalities and antisocial behavior: A systematic review of diffusion tensor imaging studies across development. NeuroImage Clin. (2017) 14:201–15. doi: 10.1016/j.nicl.2017.01.014 PMC5280002 28180079

[19] Yu T Pei W Xu C Zhang X Deng C. Prediction of violence in male schizophrenia using sMRI, based on machine learning algorithms. BMC Psychiatry. (2022) 22:676. doi: 10.1186/s12888-022-04331-1 36320010 PMC9628088

[20] Yang Y Raine A Han CB Schug RA Toga AW Narr KL. Reduced hippocampal and parahippocampal volumes in murderers with schizophrenia. Psychiatry Res. (2010) 182:9–13. doi: 10.1016/j.pscychresns.2009.10.013 20227253 PMC2855857

[21] Kuroki N Kashiwagi H Ota M Ishikawa M Kunugi H Sato N. Brain structure differences among male schizophrenic patients with history of serious violent acts: an MRI voxel-based morphometric study. BMC Psychiatry. (2017) 17:105. doi: 10.1186/s12888-017-1263-9 28327107 PMC5361832

[22] Shen D Li Q Liu J Liao Y Li Y Gong Q. The deficits of individual morphological covariance network architecture in schizophrenia patients with and without violence. Front Psychiatry. (2021) 12:777447. doi: 10.3389/fpsyt.2021.777447 34867559 PMC8634443

[23] Narayan VM Narr KL Kumari V Woods RP Thompson PM Toga AW. Regional cortical thinning in subjects with violent antisocial personality disorder or schizophrenia. Am J Psychiatry. (2007) 164:1418–27. doi: 10.1176/appi.ajp.2007.06101631 PMC3197838 17728428

[24] Hoptman MJ Antonius D Mauro CJ Parker EM Javitt DC. Cortical thinning, functional connectivity, and mood-related impulsivity in schizophrenia: relationship to aggressive attitudes and behavior. Am J Psychiatry. (2014) 171:939–48. doi: 10.1176/appi.ajp.2014.13111553 PMC4178944 25073506

[25] Baumann PS Klauser P Griffa A Golay P Palix J Alameda L. Frontal cortical thickness correlates positively with impulsivity in early psychosis male patients. Early Interv Psychiatry. (2019) 13:848–52. doi: 10.1111/eip.2019.13.issue-4 29770569

[26] Wang X Chen W Gou M Li W Li N Tong J. Relationship between plasma TNF-α levels and agitation symptoms in first episode patients with schizophrenia. BMC Psychiatry. (2024) 24:480. doi: 10.1186/s12888-024-05796-y 38956509 PMC11218378

[27] Shi C Kang L Yao S Ma Y Li T Liang Y. The MATRICS consensus cognitive battery (MCCB): co-norming and standardization in China. Schizophr Res. (2015) 169:109–15. doi: 10.1016/j.schres.2015.09.003 PMC4916953 26441005

[28] Zhang H Wang Y Hu Y Zhu Y Zhang T Wang J. Meta-analysis of cognitive function in Chinese first-episode schizophrenia: MATRICS Consensus Cognitive Battery (MCCB) profile of impairment. Gen Psychiatr. (2019) 32:e100043. doi: 10.1136/gpsych-2018-100043 31423473 PMC6677937

[29] Šneidere K Zdanovskis N Mondini S Stepens A. Relationship between lifestyle proxies of cognitive reserve and cortical regions in older adults. Front Psychol. (2023) 14:1308434. doi: 10.3389/fpsyg.2023.1308434 38250107 PMC10797127

[30] Laviolette SR Grace AA. The roles of cannabinoid and dopamine receptor systems in neural emotional learning circuits: implications for schizophrenia and addiction. Cell Mol Life Sci. (2006) 63:1597–613. doi: 10.1007/s00018-006-6027-5 PMC11136137 16699809

[31] Whitfield-Gabrieli S Thermenos HW Milanovic S Tsuang MT Faraone SV McCarley RW. Hyperactivity and hyperconnectivity of the default network in schizophrenia and in first-degree relatives of persons with schizophrenia. Proc Natl Acad Sci U.S.A. (2009) 106:1279–84. doi: 10.1073/pnas.0809141106 PMC2633557 19164577

[32] Calabrese DR Wang L Harms MP Ratnanather JT Barch DM Cloninger CR. Cingulate gyrus neuroanatomy in schizophrenia subjects and their non-psychotic siblings. Schizophr Res. (2008) 104:61–70. doi: 10.1016/j.schres.2008.06.014 18692994 PMC4256942

[33] Athanassiou M Dumais A Tikasz A Lipp O Dubreucq JL Potvin S. Increased cingulo-orbital connectivity is associated with violent behaviours in schizophrenia. J Psychiatr Res. (2022) 147:183–9. doi: 10.1016/j.jpsychires.2022.01.001 35051717

[34] Cui LB Wang XY Fu YF Liu XF Wei Y Zhao SW. Transcriptional level of inflammation markers associates with short-term brain structural changes in first-episode schizophrenia. BMC Med. (2023) 21:250. doi: 10.1186/s12916-023-02963-y 37424013 PMC10332052

[35] Cheng N Ma H Zhang K Zhang C Geng D. The predictive value of monocyte/high-density lipoprotein ratio (MHR) and positive symptom scores for aggression in patients with schizophrenia. Medicina (Kaunas). (2023) 59(3):503. doi: 10.3390/medicina59030503 36984504 PMC10055014

[36] Hoang D Xu Y Lutz O Bannai D Zeng V Bishop JR. Inflammatory subtypes in antipsychotic-naïve first-episode schizophrenia are associated with altered brain morphology and topological organization. Brain Behav Immun. (2022) 100:297–308. doi: 10.1016/j.bbi.2021.11.019 34875344 PMC8767408

[37] Zhou C Zhang R Ding M Duan W Fang J Tang X. Progressive structural alterations associated with negative symptoms in schizophrenia: A causal structural covariance network analysis. Prog Neuropsychopharmacol Biol Psychiatry. (2025) 136:111236. doi: 10.1016/j.pnpbp.2024.111236 39732315

[38] Wan X Cheng K Tanaka K. Neural encoding of opposing strategy values in anterior and posterior cingulate cortex. Nat Neurosci. (2015) 18:752–9. doi: 10.1038/nn.3999 25894290

[39] Bueso-Izquierdo N Verdejo-Román J Contreras-Rodríguez O Carmona-Perera M Pérez-García M Hidalgo-Ruzzante N. Are batterers different from other criminals? fMRI study. Soc Cognit Affect Neurosci. (2016) 11:852–62. doi: 10.1093/scan/nsw020 PMC4847704 26884544

[40] Holt DJ Cassidy BS Andrews-Hanna JR Lee SM Coombs G Goff DC. An anterior-to-posterior shift in midline cortical activity in schizophrenia during self-reflection. Biol Psychiatry. (2011) 69:415–23. doi: 10.1016/j.biopsych.2010.10.003 PMC3740539 21144498

[41] Zhang H Wei X Tao H Mwansisya TE Pu W He Z. Opposite effective connectivity in the posterior cingulate and medial prefrontal cortex between first-episode schizophrenic patients with suicide risk and healthy controls. PloS One. (2013) 8:e63477. doi: 10.1371/journal.pone.0063477 23704911 PMC3660523

[42] Ross MC Cisler JM. Altered large-scale functional brain organization in posttraumatic stress disorder: A comprehensive review of univariate and network-level neurocircuitry models of PTSD. NeuroImage Clin. (2020) 27:102319. doi: 10.1016/j.nicl.2020.102319 32622316 PMC7334481

[43] Sun Q Zhang Y Zhou J Wang X. Altered resting-state functional connectivity in the default mode network in male juvenile violent offenders. Brain Imaging Behav. (2022) 16:608–16. doi: 10.1007/s11682-021-00535-3 PMC9010331 34480692

[44] Meyer-Lindenberg A Buckholtz JW Kolachana B Hariri RA Pezawas L Blasi G. Neural mechanisms of genetic risk for impulsivity and violence in humans. Proc Natl Acad Sci U.S.A. (2006) 103:6269–74. doi: 10.1073/pnas.0511311103 PMC1458867 16569698

[45] Kim SY Kaufman MJ Cohen BM Jensen JE Coyle JT Du F. *In vivo* brain glycine and glutamate concentrations in patients with first-episode psychosis measured by echo time-averaged proton magnetic resonance spectroscopy at 4T. Biol Psychiatry. (2018) 83:484–91. doi: 10.1016/j.biopsych.2017.08.022 PMC5809251 29031411

[46] Ichinose M Park S. Mechanisms underlying visuospatial working memory impairments in schizophrenia. Curr Top Behav Neurosci. (2019) 41:345–67. doi: 10.1007/7854_2019_99 31407240

[47] Elton A Gao W. Task-positive functional connectivity of the default mode network transcends task domain. J Cognit Neurosci. (2015) 27:2369–81. doi: 10.1162/jocn_a_00859 26244722

[48] Xue K Chen J Wei Y Chen Y Han S Wang C. Impaired large-scale cortico-hippocampal network connectivity, including the anterior temporal and posterior medial systems, and its associations with cognition in patients with first-episode schizophrenia. Front Neurosci. (2023) 17:1167942. doi: 10.3389/fnins.2023.1167942 37342466 PMC10277613

[49] Zhang Z Yan T Wang Y Zhang Q Zhao W Chen X. Polymorphism in schizophrenia risk gene MIR137 is associated with the posterior cingulate Cortex’s activation and functional and structural connectivity in healthy controls. NeuroImage Clin. (2018) 19:160–6. doi: 10.1016/j.nicl.2018.03.039 PMC6051762 30035013

[50] Tamnes CK Herting MM Goddings AL Meuwese R Blakemore SJ Dahl RE. Development of the cerebral cortex across adolescence: A multisample study of inter-related longitudinal changes in cortical volume, surface area, and thickness. J Neurosci. (2017) 37:3402–12. doi: 10.1523/JNEUROSCI.3302-16.2017 PMC5373125 28242797

[51] Thormodsen R Jensen J Holmèn A Juuhl-Langseth M Emblem KE Andreassen OA. Prefrontal hyperactivation during a working memory task in early-onset schizophrenia spectrum disorders: an fMRI study. Psychiatry Res. (2011) 194:257–62. doi: 10.1016/j.pscychresns.2011.05.011 22079661

